# Periodic Assessment of Trajectories of Housing, Homelessness, and Health Study (PATHS): Protocol for a Prospective Cohort Study of People Experiencing Homelessness

**DOI:** 10.2196/74266

**Published:** 2025-09-04

**Authors:** Randall Kuhn, Jessie Chien, Norma Guzman Hernandez, Taylor M Mobley, Danielle Paulazzo, Gisele Corletto, Benjamin F Henwood

**Affiliations:** 1 Department of Community Health Sciences Fielding School of Public Health University of California, Los Angeles Los Angeles, CA United States; 2 Department of Epidemiology Fielding School of Public Health University of California, Los Angeles Los Angeles, CA United States; 3 Suzanne Dworak-Peck School of Social Work University of Southern California Los Angeles, CA United States

**Keywords:** unsheltered homelessness, housing instability, digital data collection, mobile phone surveys, longitudinal study, homeless health risks

## Abstract

**Background:**

The past decade has seen a substantial increase in the number of people experiencing unsheltered homelessness. The unsheltered population faces heightened health and social risks, yet research on their experiences remains limited.

**Objective:**

This paper presents the protocol for the Periodic Assessment of Trajectories of Housing, Homelessness, and Health Study (PATHS), a longitudinal study that leverages mobile phone technology and web-based surveys to track the housing and health trajectories of people experiencing unsheltered homelessness in Los Angeles County.

**Methods:**

Participants were recruited from the Los Angeles County Homeless Count Demographic Survey, an annual representative survey of the county’s unsheltered population. Eligibility criteria included being aged ≥18 years, having stayed in an unsheltered location or homeless shelter for at least 1 night in the past month, and residing in Los Angeles County. The study uses a web-based survey platform accessible via mobile phones and provides electronic gift card incentives for participation. Data on housing, health, and social outcomes are collected monthly using trauma-informed, equity-sensitive surveys, designed for diverse literacy levels with a user-friendly interface that includes buffers for sensitive topics.

**Results:**

Since the study launched in December 2021, a total of 2058 individuals have been screened and found eligible. In total, 57.43% (n=1182) of participants completed the baseline survey, of whom 75.47% (n=892) completed at least 1 monthly survey. By December 2024, participants had contributed 7585 monthly surveys (average of 8.5, SD 8.36 per respondent and median of 6, IQR 2-11). Compared to the unsheltered population of Los Angeles County, the PATHS sample overrepresents younger adults aged <40 years (641/1182, 54.23% vs 38.64%) and female participants (507/1182, 42.89% vs 27.74%). Furthermore, the PATHS cohort reports a high burden of health risks relative to the housed population, with 47.3% (422/892) reporting symptoms of anxiety (vs 19.1%), 45.1% (402/892) reporting symptoms of depression (vs 16.4%), 35% (312/892) reporting a disability (vs 12.9%), and 69.4% (619/892) experiencing food insecurity (vs 15.7%).

**Conclusions:**

PATHS offers an innovative platform for real-time monitoring of the housing, health, and service needs of people experiencing unsheltered homelessness in Los Angeles County. By leveraging continuous, in-depth data collection via mobile surveys, PATHS provides valuable insights into the evolving challenges faced by this population. Addressing critical gaps in longitudinal research, PATHS has the potential to drive more informed policy decisions and interventions that improve outcomes for this population considered vulnerable.

**International Registered Report Identifier (IRRID):**

DERR1-10.2196/74266

## Introduction

### Background

Over the past decade, the number of people experiencing homelessness in the United States has increased substantially [1]. While people experiencing homelessness is widely used as a person-first term in academic and policy contexts, we recognize that some individuals with lived experience prefer terms such as “homeless” or “unhoused.” We intend to use respectful, person-centered language while aligning with terminology commonly used in the field. Unsheltered homelessness—defined as sleeping on the streets, in vehicles, in tents, or in other places not meant for human habitation—is a growing phenomenon within this crisis, particularly in the Western United States [1,2]. According to the Point-in-Time Count by the US Department of Housing and Urban Development, the estimated number of unsheltered people experiencing homelessness on a single night in January between 2014 and 2024 rose by 56%, from 175,399 to 274,224 [1].

People experiencing homelessness face numerous health risks, including harassment, physical injury, poor sanitation, inadequate nutrition, and limited health care access [3-7], contributing to higher rates of chronic diseases (eg, obesity, cancer, and depression), earlier onset of geriatric conditions, and a 20-year shorter life expectancy compared to the general population [8-11]. Exposure to unsheltered homelessness is associated with even greater social and health challenges, such as poor sleep, extreme weather, food insecurity, policing, exposure to violence, and substance use, which are likely to further accelerate premature aging and mortality [3,4,9,12-15]. A recent literature review found that most existing studies pointed to associations between unsheltered homelessness and poorer health outcomes compared to those who were sheltered [12]. However, the review also highlighted that few longitudinal studies track the well-being of people experiencing homelessness over time [16,17], whether sheltered or unsheltered, and none have done so at time intervals that highlight the ways in which their lives can change in a short period.

Los Angeles (LA) County is home to the largest population of unsheltered people experiencing homelessness in the United States [18]. In 2024, LA County had approximately 75,000 people experiencing homelessness in a single night, with almost 70% living unsheltered [18], a figure that far exceeds those seen in other major US cities and global urban centers such as London and Tokyo [19]. For comparison, New York City reported approximately 140,000 people experiencing homelessness, but the vast majority (97%) were sheltered [20]. A significant driver of LA County’s unsheltered crisis is its jail system, the largest in the world, which has become a central part of an institutional cycle involving homelessness, poverty, substance use, and mental illness [21,22]. Decades of underinvestment in behavioral health services, coupled with rising housing costs, have forced thousands of individuals with untreated psychiatric conditions to live on the streets [22]. In the absence of adequate support systems, jails have become shelters of last resort for many with severe psychiatric needs [22-24]. Upon release, individuals often fall into a revolving door of incarceration and street homelessness, as criminal records create substantial barriers to employment, housing, and public services, further entrenching the conditions that led to their incarceration [22,24]. The convergence of housing insecurity, untreated mental illness, and mass incarceration reflects a systemic breakdown in the health care, housing, and social service systems—one that continues to fail the county’s residents experiencing the greatest vulnerability [22,24].

In the past 5 years, the county has invested in various interventions aimed at reducing the size of its unsheltered population, including new supportive services and permanent supportive housing programs [25-28]. However, the lack of real-time data collection of the housing and service needs, as well as other basic metrics of well-being among unsheltered individuals, has hindered the effective delivery of these efforts. Furthermore, amid growing public concerns about livability, LA County, similar to many municipalities nationwide, has introduced new camping ordinances and ramped up efforts to remove homeless encampments through “sweeps” [29,30]. These actions often combine policing with efforts to connect individuals to housing and services [30]. The growing reliance on these more punitive approaches to homelessness management raises concerns about whether these strategies can help individuals transition into stable housing, or whether sweeps merely displace people experiencing homelessness, disrupt their social networks, and expose them to further health risks [31]. Timely tracking of these outcomes can help assess policy impact and inform program development.

The *Periodic Assessment of Trajectories of Housing, Homelessness, and Health Study* (PATHS) aims to address this knowledge gap by following a cohort of predominantly unsheltered people experiencing homelessness in LA County. PATHS overcomes previous challenges of conducting longitudinal research with this highly mobile population by leveraging their widespread use of mobile phones to administer monthly web-based surveys. Mobile phones have become an increasingly important tool in social and behavioral research due to their low cost, ease of use, customizable applications, built-in sensors (eg, GPS and accelerometer), and ubiquity across socioeconomic groups [32,33]. In a sample of people experiencing homelessness in LA County, >90% of adults owned and used mobile phones daily [34]. Mobile phone surveys can be delivered remotely and completed by participants independently, allowing them to respond at their convenience within scheduled time frames. This flexibility makes mobile phones an invaluable tool for capturing the challenges faced by people experiencing homelessness with the frequency needed to track the rapid unfolding of critical health and socioeconomic events.

### Objectives

This paper reports on our efforts to develop a multipurpose data collection platform designed to enable an action-learning approach to addressing the housing, health, and service needs of people experiencing unsheltered homelessness. We describe our recruitment methodology and the challenges of implementing the study using trauma-informed design principles. In addition, we present initial findings from follow-up surveys, highlighting areas of monthly consistency and variability, which underscore the advantages of a monthly survey model. We conclude by reflecting on the limitations and strengths of our approach, as well as the potential opportunities to expand its reach and impact.

## Methods

### Study Design Overview

PATHS is a longitudinal cohort study of adults experiencing homelessness who were unsheltered at the time of recruitment in LA County. Using a web-based survey platform and electronic gift card incentives, the study collects recurring data on a broad range of health, housing, and social outcomes. With an ongoing panel, we also regularly update the study questionnaires to incorporate new questions that evaluate emerging issues and initiatives affecting LA County’s unsheltered population, such as changes in rehousing efforts, policing, and health care delivery. Participation involves a screening survey, a baseline survey, monthly surveys, and biannual follow-up surveys, distributed in January and July of each year. All survey distribution and data collection are carried out through Qualtrics (Qualtrics International, Inc), a secure platform that meets US federal standards for protecting personal health information as outlined in the Health Insurance Portability and Accountability Act of 1996 [35].

The study is conducted collaboratively by investigators from the University of California, Los Angeles (UCLA) Fielding School of Public Health and the University of Southern California (USC) Dworak-Peck School of Social Work, with human subject protection overseen by the UCLA Institutional Review Board. The study builds on an earlier pilot study in which 136 people experiencing homelessness, who were patients of a Federally Qualified Health Center, were enrolled electronically in a monthly survey, generating actionable research on COVID-19 vaccine uptake and retaining 66% of clients for 3 months [36].

PATHS was codeveloped in collaboration with individuals with lived experience of unsheltered homelessness, who contributed to the survey design and content to ensure cultural relevance. PATHS results are regularly interpreted by lived experts via presentations conducted through the Homelessness Policy Research Institute at the University of Southern California and other community presentations. These sessions have helped to ensure that findings accurately represent the challenges faced by people experiencing homelessness and are accessible and actionable for both people experiencing homelessness and service providers. Throughout the study period, we will solicit feedback more intensively through community presentations and by reporting key findings back to respondents themselves. Insights from these engagements will inform ongoing study improvements and guide the development of dissemination materials.

### Recruitment

Launched in December 2021, PATHS has completed 3 waves of annual recruitment, primarily through LA County’s annual Homeless Count Demographic Survey (DS), which accompanies the county’s official point-in-time count of people experiencing homelessness, as mandated by the US Department of Housing and Urban Development. The DS collects data from a sample of adults experiencing homelessness in unsheltered settings to estimate the characteristics of this population across the county and the number of people living in cars, vans, recreational vehicles, tents, and makeshift shelters captured in the point-in-time count [37].

Each year from December to March, the DS field team deploys approximately 25 trained surveyors to canvas around 500 randomly selected census tracts across the county and administers a short, 1-time DS to approximately 5000 unsheltered adults. Detailed methods are documented in LA County’s annual Homeless Count reports [37]. Briefly, DS sampling occurs in 25% of the county’s census tracts, using a sample drawn from hot spots and non–hot spots via Neyman allocation. All DS estimates are weighted to account for tract selection, nonresponse, and refusals. Enumerators are trained to collect “perceived” data on age, sex, race or ethnicity, and health status for both surveyed and nonsurveyed individuals. Nonrespondents are represented by the most similar respondents based on nearest neighbor weighting, which accounts for perceived characteristics and spatial proximity. Estimates for the DS are calculated with and without these weights and are closely aligned. Unsheltered adults who completed the DS are invited to participate in PATHS; in 2024, approximately 37.47% (1605/4283) of DS respondents accepted the invitation. Additional recruitment for the study has been conducted through dedicated canvassing in hot spots of unsheltered homelessness annually between April and July.

To enroll, participants must have a valid mobile phone number at the time of recruitment to check for previous enrollment and receive the initial screening survey. Individuals interested in participating are enrolled in 1 of two ways: (1) surveyors collect their mobile phone numbers through a webform, after which participants receive an SMS text message with a link to the study enrollment website on Qualtrics via SMS text message, or (2) surveyors distribute printed study cards with a QR barcode and phone number, allowing prospective participants to scan the code or SMS text message the number to access the study website (Multimedia Appendix 1). The enrollment website provides information about the study and then guides participants through the self-administered informed consent process (Multimedia Appendix 2). Once consent is obtained, participants fill out a brief screening survey to determine whether they meet the study’s eligibility criteria: (1) have lived in a homeless shelter or unsheltered setting (eg, street, vehicle, tent, or makeshift shelter) for at least 1 night in the past month, (2) live in LA County, and (3) be aged ≥18 years. Continued participation also requires a valid email address to receive electronic gift card incentives. Eligible participants are sent the baseline survey within 2 days of being screened.

Once the baseline survey is completed, participants receive links to follow-up surveys each month via SMS text message or email. All surveys are self-administered over the Qualtrics web interface, accessible via phone or computer, and are available in both English and Spanish.

### Ethical Considerations

Ethics approval for the study was granted by the UCLA Institutional Review Board (IRB#21-001148) in September 2021. As noted in the Recruitment section, informed consent was obtained from all participants through a self-administered, online consent process after the nature and potential consequences of the study were explained and before eligibility screening. Ethical standards were maintained for all digital data collection tools used in the study.

Respondents initially received a US $5 financial incentive for each completed survey, which was raised to US $10 per survey in February 2022, and subsequently to US $20 per survey in August 2024. Incentives are provided in the form of an electronic gift card sent directly to their email, which can be redeemed at their choice from among 8 local retail locations. As of December 2024, the gift card redemption rate is approximately 90%.

All reasonable steps are taken to protect the privacy and confidentiality of research participants. Each individual is assigned a unique identifier that is separate from any personally identifiable information (ie, mobile phone numbers and email addresses), ensuring that all study data are deidentified. Raw survey data, including personally identifiable information, remains securely stored on the Qualtrics platform, compliant with the Health Insurance Portability and Accountability Act, until transferred to a password-protected encrypted server. Before any analysis, the data are deidentified and stored in a restricted-access folder on the research server, accessible only to select research staff with appropriate training in research ethics, compliance, and data security.

### Retention Strategies

To enhance response rates, starting in March 2023, participants are asked after each survey to indicate their preferred method of contact for future surveys. They can choose to receive subsequent surveys via SMS text message or email, with the option to switch between modes each month based on their preference. In addition, we provide a dedicated hotline, available via email, SMS text message, or phone call, for participants to contact if they experience any issues accessing their surveys or receiving their incentives. Furthermore, to address nonresponse due to changing phone numbers, we recontact participants lost to follow-up (ie, those who have not completed the last 3 monthly surveys) via the email address used for their electronic gift card, allowing them to update their contact information or switch their preferred mode of contact.

### Measures

PATHS collects data on various topics related to people’s housing and health experiences and the social determinants of health through multiple surveys. The initial surveys were developed and tested by people with lived experience of homelessness to ensure that they were trauma-informed, equity-sensitive, and appropriate to a broad range of literacy levels. In addition, the surveys feature a visually engaging interface to encourage completion and incorporate techniques such as buffers to prepare respondents for potentially difficult, sensitive, or triggering questions (Multimedia Appendix 3).

The core measures collected in this study as of December 2024 are listed in Table 1. The baseline survey captures sociodemographic characteristics and homelessness history. The baseline survey and regular biannual follow-up surveys, conducted in January and July of each year, collect self-reported data on health diagnoses, health care access, social services use, housing and health needs, and disability status. The monthly surveys cover topics related to housing status; service engagement; access to basic necessities; policing; discrimination or violence; and physical, social, and mental well-being. Periodic survey modules have been updated to address the rapidly changing ecosystem of service, policing, and environmental risks, including events such as the wildfires that hit LA County in January 2025. Furthermore, the monthly survey collects participants’ geographic location, which they can provide either by sharing exact coordinates through their devices or by self-reporting the address, cross streets, or neighborhood where they are staying, which can be linked to administrative spatial data.

**Table 1:** Study measures.

Domains		Measures and their description
**Baseline and biannual follow-up surveys**		
	Sociodemographics (baseline only)	Age, sex at birth, gender identity, sexual orientation, race or ethnicity, veteran status, educational attainment, and history of criminal legal system involvement
	Homelessness history (baseline only)	Duration
	Self-reported health diagnoses	Chronic physical health conditions (eg, serious heart condition, cardiovascular disease, diabetes, HIV or AIDS, cancer, immunocompromised status, asthma, chronic respiratory disease, chronic kidney disease, blood disorder, liver disease, neurodegenerative disease, and arthritis), neuropsychiatric disorders (eg, major depression, anxiety, bipolar disorder, schizophrenia or other psychotic disorder, personality disorder, and posttraumatic stress disorder), substance use disorders (eg, alcohol use, cannabis or marijuana use, stimulant use, and opioid use), and history of substance use treatment
	Coping strategies	PC-PTSD-5^a^, 6-item BRS^b^, Insignia Patient Activation Measure (4 items), and DERS^c^
	Access to resources and services	Health insurance coverage, receipt of government benefits (eg, SSI^d^ or SSDI^e^, SNAP^f^, and unemployment), have a usual source of care, and MNA-SF^g^
	Health care needs	Health conditions of greatest concern and quality of care received for the condition, as well as trust in the medical profession [38]
	Housing needs	Preferences for types of shelter or housing (eg, safe camping, congregate, bridge housing, and supportive housing), housing needs (eg, storage and accommodations for pets, spouse, and child), and housing concerns (eg, hours or curfew, cleanliness, and safety)
	Disability	ACS-6^h^ on disability status
**Monthly surveys**		
	Physical health	Self-rated physical health (1 item, PROMIS^i^ Global Health Scale version 1.2) and body pain (1 item, Medical Outcomes Study Physical Functioning Scale)
	Psychosocial health	PHQ-4^j^ for anxiety and depression, PROMIS Sleep Disturbance Scale (2 items), and 5-item UCLA^k^ Loneliness Scale
	Substance use	AUDIT-C^l^, WHO ASSIST^m^, overdose, and receipt of treatment
	Basic needs	USDA^n^ Food Security Module (3 items) and access to sanitation and personal hygiene facilities (ie, clean drinking water, toilets, and showers)
	Health care use	Emergency room visits, hospitalizations, unmet health care needs (ever wanted or needed health care but did not obtain it), medication adherence, and type of health care services received
	Homeless services engagement	Offers of shelter or housing and contact with a homeless services outreach worker
	Housing status	Housing status (last night, at any point in the past month, and most often in the past month), perceptions of safety, and displacement or changes in sleeping locations
	Policing and criminal legal system involvement	Police interaction, arrests, move-along citation, exposure to encampment sweeps, and incarceration
	Violence and discrimination	National Crime Victimization Survey (2 items: assault and harassment) and Everyday Discrimination Scale (short version, 5 items)
	Employment	Work history and income

^a^PC-PTSD-5: Primary care posttraumatic stress disorder screen for Diagnostic and Statistical Manual of Mental Disorders, Fifth Edition.

^b^BRS: Brief Resilience Scale.

^c^DERS: Difficulties in Emotional Regulation Questionnaire.

^d^SSI: Supplemental Security Income.

^e^SSDI: Social Security Disability Insurance.

^f^SNAP: Supplemental Nutrition Assistance Program.

^g^MNA-SF: Mini Nutritional Assessment–Short Form.

^h^ACS-6: 6-item American Community Survey.

^i^PROMIS: Patient-Reported Outcomes Measurement Information System.

^j^PHQ-4: 4-item Patient Health Questionnaire.

^k^UCLA: University of California, Los Angeles.

^l^AUDIT-C: Alcohol Use Disorders Identification Test–Consumption.

^m^WHO ASSIST: World Health Organization Alcohol, Smoking, and Substance Involvement Screening Test.

^n^USDA: United States Department of Agriculture.

## Results

This project is funded by the Conrad N Hilton Foundation, with funding awarded in September 2021. Data collection began in December 2021 and is ongoing. Data analysis is performed on a rolling basis to address specific research questions and stakeholder priorities as they emerge.

### Recruitment

**Figure 1:**
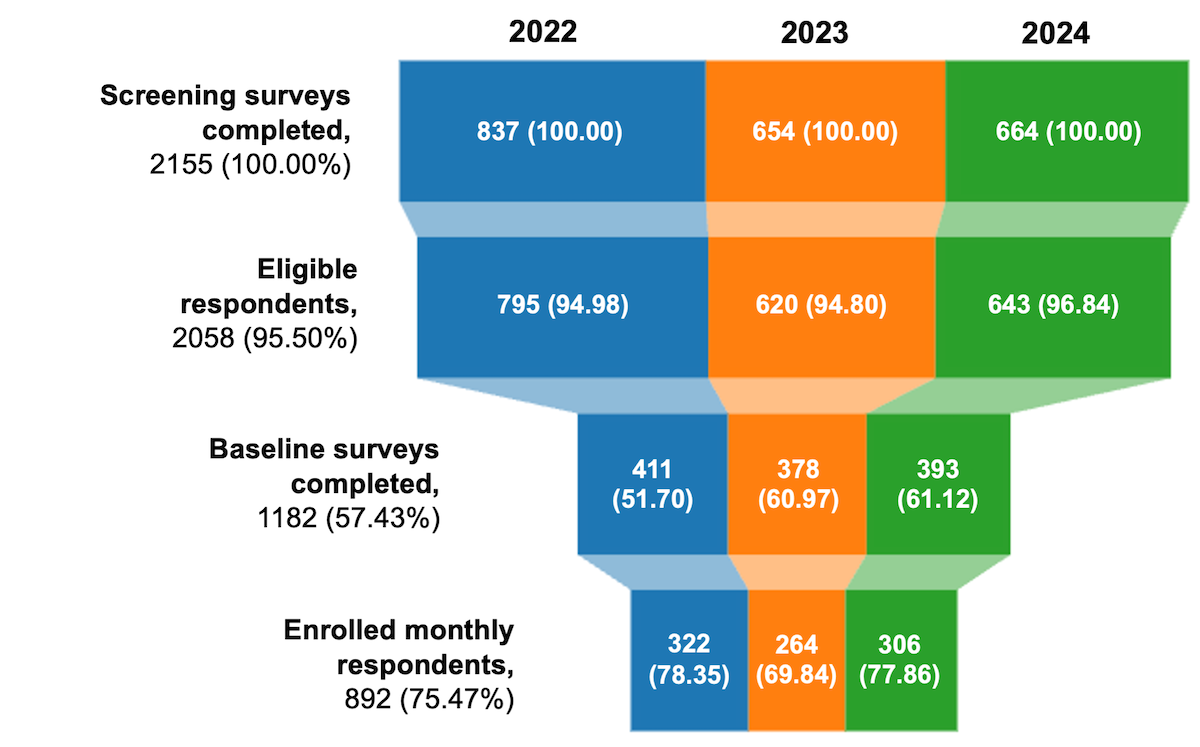
Periodic Assessment of Trajectories of Housing, Homelessness, and Health Study screening and enrollment funnel, by recruitment year (2022 to 2024). Percentages are calculated using the number of participants in the previous stage as the denominator.

Figure 1 shows screening and enrollment statistics for 3 years of PATHS recruitment, covering the period from December 2021 to July 2024. Among the 2155 participants screened throughout the study period, 2058 (95.49%) met the eligibility criteria, with the majority (1840/2058, 89.41%) having spent at least 1 night in an unsheltered location. A total of 1182 (57.43%) participants completed the baseline survey, of whom 892 (75.47%) continued to the monthly survey, officially enrolling in the study. As of December 1, 2024, enrolled participants have contributed a total of 7585 monthly surveys (average of 8.5 surveys per respondent). In the most recent 6-month follow-up period (June to December 2024), 416 (46.6%) of the enrolled participants responded to at least 1 monthly survey (herein referred to as the “recent monthly follow-up sample”).

### Study Population

To examine the representativeness of the PATHS sample to LA County’s unsheltered population, Table 2 compares the sociodemographic characteristics of the PATHS baseline sample and recent monthly follow-up (between July and December 2024) sample respondents to respondents of the LA County Homeless Count DS from 2022 to 2024, which is considered to characterize LA County’s unsheltered population.

**Table 2:** Comparison of the Periodic Assessment of Trajectories of Housing, Homelessness, and Health Study (PATHS) samples to Los Angeles (LA) County’s unsheltered population, 2022 to 2024.

Characteristic		2022 to 2024 LA County unsheltered population^a^ (%)	PATHS baseline sample (n=1182), n (%)	PATHS recent monthly follow-up sample^b^ (n=416), n (%)
**Age (y)**				
	18-24	4.49	105 (8.88)	49 (11.78)
	25-39	34.15	536 (45.35)	176 (42.31)
	40-49	23.94	267 (22.59)	99 (23.80)
	50-59	21.78	194 (16.41)	60 (14.42)
	≥60	15.64	80 (6.77)	32 (7.69)
**Sex**				
	Male	69.14	635 (53.72)	203 (48.80)
	Female	27.74	507 (42.89)	199 (47.84)
	Neither male nor female	3.12	40 (3.38)	14 (3.37)
**Race or ethnicity**				
	Hispanic or Latinx	40.67	413 (34.94)	169 (40.63)
	Non–Hispanic American Indian or Alaskan Native	1.58	12 (1.02)	6 (1.44)
	Non–Hispanic Asian	1.33	18 (1.52)	9 (2.16)
	Non–Hispanic Black or African American	28.59	315 (26.65)	96 (23.08)
	Non–Hispanic Native Hawaiian or Pacific Islander	0.40	8 (0.68)	1 (0.24)
	Non–Hispanic White	23.97	325 (27.50)	103 (24.76)
	Others or unknown^c^	3.46	91 (7.70)	32 (7.69)
Homeless for >1 y		79.33	947 (80.12)	321 (77.16)
Past treatment for substance use disorder		31.79	372 (31.47)	97 (23.37)

^a^Values for LA County’s unsheltered population come from the LA Homeless Count Demographic Survey of adults experiencing homelessness, combined for the years 2022, 2023, and 2024. Approximately 5000 unsheltered individuals in LA are surveyed each year for the Homeless Count. Absolute counts are not reported because values have been adjusted using statistical weighting to improve representativeness and account for sampling design.

^b^PATHS participants who responded to at least 1 monthly survey in the last 6 months.

^c^Percentages for the PATHS samples include respondents who identified as multiracial.

Compared to the county’s unsheltered population, participants in the baseline and recent monthly follow-up PATHS samples were slightly younger (641/1182, 54.23% aged <40 y in the baseline sample, 225/416, 54.1% aged <40 y in the recent monthly follow-up sample, vs 38.64% in the county unsheltered population), which may reflect differences between age groups in mobile phone access and technology literacy needed to enroll in the study and consistently respond to the monthly surveys. PATHS also had higher rates of participation in the baseline survey among female individuals than the overall unsheltered population (507/1182, 42.89% vs 27.74%); this overrepresentation was even more pronounced in the recent monthly follow-up sample (199/416, 47.8% vs 27.74%). However, higher female individual participation rates are common in many volunteer-based opinion surveys, given the distinct challenges faced by women experiencing homelessness [39,40]. This response pattern allows us to better examine their specific needs. In comparison to the county’s overall unsheltered population, Hispanic or Latinx individuals were underrepresented in the baseline sample (413/1182, 34.94% vs 40.67%), while non–Hispanic White individuals were slightly overrepresented (325/1182, 27.5% vs 23.97%). However, these differences became much more comparable in the recent monthly follow-up sample. On the other hand, non–Hispanic Black individuals were underrepresented in the recent monthly follow-up sample compared to the overall unsheltered population, highlighting differences in retention rates across racial groups. Both samples mirrored the unsheltered population in homelessness duration (ie, having been homeless for >1 y). Finally, a higher proportion of individuals with a history of substance use treatment were recruited into the study compared to the county’s unsheltered population. However, this group experienced a drop in the recent monthly follow-up sample, likely due to challenges in retaining individuals facing the complex intersection of homelessness and substance use.

### Retention

To examine participant retention, Table 3 displays the response rate to the monthly surveys during the first 6 calendar months following enrollment: overall and stratified by recruitment year. Overall, 39.2% (350/892) of enrolled participants completed most (5 to 6) of the monthly surveys distributed, while just less than a quarter (205/892, 23%) of participants did not provide any responses after enrollment. Response rates were generally higher among participants recruited in 2023 and 2024 compared to those recruited in 2022. This improvement may be attributed to our decision to allow participants to choose their preferred method for receiving the survey link, either via SMS text message or email.

**Table 3:** Response rate to the monthly surveys during the first 6 months after enrollment, by recruitment year.

Respondents	Overall (n=892), n (%)	By recruitment year, n (%)		
		2022 (n=322)	2023 (n=264)	2024 (n=306)
0%	205 (23)	89 (27.6)	50 (18.9)	66 (21.6)
0%-34%	193 (21.6)	69 (21.4)	57 (21.6)	67 (21.9)
34%-67%	144 (16.1)	47 (14.6)	43 (16.3)	54 (17.6)
67%-100%	350 (39.2)	117 (36.3)	114 (43.2)	119 (38.9)

### Population Health

Table 4 [41-43] characterizes the extraordinarily high burden of poor health and health risk among the entire PATHS monthly survey respondent sample, compared to the housed population of LA County. Almost half (441/892, 49.4%) of the PATHS sample rated their health as fair or poor (vs 16.9% of LA County adults), 47.3% (422/892) had symptoms of anxiety (vs 19.1%), 45.1% (402/892) had symptoms of depression (vs 16.4%), 36.7% (327/892) smoked cigarettes regularly (vs 5.2%), and 35% (312/892) reported having a disability (vs 12.9%). Food insecurity was particularly severe: 69.4% (619/892) of PATHS monthly respondents reported experiencing food insecurity, which is 4 times as high as the rate in the adult population of LA County (15.7%).

**Table 4:** Comparison of selected health indicators among the Periodic Assessment of Trajectories of Housing, Homelessness, and Health Study (PATHS) monthly respondent sample to the Los Angeles County adult population.

Indicator	PATHS monthly survey respondents (n=892), n%	Los Angeles County’s adult population, %
Poor of self-rated	441 (49.4)	16.9
Symptoms of anxiety (GAD-2^a^)	422 (47.3)	19.1
Symptoms of depression (PHQ-2^b^)	402 (45.1)	16.4
Regular smoking	327 (36.7)	5.2
Food insecurity	619 (69.4)	15.7
Any disability	312 (35.0)	12.9

^a^GAD-2: 2-item Generalized Anxiety Disorder.

^b^PHQ-2: 2-item Patient Health Questionnaire.

## Discussion

### Anticipated Findings

PATHS addresses multiple gaps in homelessness research and policy, including a lack of representative survey-based data on unsheltered populations, a lack of longitudinal data systems for addressing housing trajectories and well-being of people experiencing homelessness, and a lack of longitudinal evaluations of interventions affecting unsheltered people experiencing homelessness [12,27]. Research from other US cities, such as New York City and Boston, has largely focused on the sheltered population [8]. While these studies offer important insights into service delivery and shelter-based interventions, they often fail to capture the scale and complexity of unsheltered homelessness seen in regions in the Western United States, such as LA, where unsheltered population rates are significantly higher [1]. People experiencing homelessness, especially those living unsheltered, can be conceptualized as part of what Castells [44] describes as the “Fourth World”: populations facing extreme marginalization within affluent societies and excluded from housing, health care, and legal protections. This exclusion gives rise to the recurring movement of people experiencing homelessness between the streets, shelters, hospitals, and jails, representing a “revolving door” pattern stemming from fragmented systems of care [45,46]. Repeated institutional contacts can contribute to the “mortification of the self,” a concept introduced by Goffman [47] in which individuals experience a loss of identity, autonomy, and social roles through constant surveillance, regulation, and disempowerment [48]. By focusing on unsheltered individuals, who remain critically underrepresented in empirical investigations, our study can provide evidence of this extreme marginalization and institutional cycling, as framed by these theoretical concepts. In doing so, we offer valuable insights into the lived, dynamic realities of this population, highlighting the transitory nature of their living situations and the daily health and social challenges they face.

Our study will offer new insights into the relationships between homelessness and health trajectories in a population-based sample—a critical remaining gap in the homelessness and health literature [49,50]. Past longitudinal studies that attempted to follow people experiencing homelessness have generated critical insights into the health experiences of homeless populations, including those transitioning out of shelter systems [10,16,17,51,52]. For instance, in a prospective cohort study of 350 homeless adults aged ≥50 years in Oakland, California, 35% reported persistent or worsening functional impairment over 3 years, compared to the 20% typically seen in the general US older adult population [53]. In a national longitudinal study of 4651 adolescents and young adults in the United States, those who had experienced homelessness faced higher risks of health-limiting conditions and poor self-rated health over an 8-year period [54]. A 2-year study in Ottawa, Canada, found that among 329 single homeless individuals, those who avoided substance use and secured high-quality housing demonstrated better mental health outcomes at follow-up [55]. In a study of 136 women experiencing homelessness in Madrid, Spain, two-thirds reported feeling discriminated against due to their housing status, and this perceived discrimination was linked to poorer mental health at follow-up [56]. Despite these findings, much of the existing longitudinal research on people experiencing homelessness used purposive sampling methods, targeted specific age groups, and lacked the sample size to make reliable inferences about subpopulations. PATHS represents a feasible approach to building on the findings of these studies by collecting monthly data more frequently and examining the impacts of diverse socioenvironmental conditions on the health trajectories of people experiencing homelessness.

Furthermore, our longitudinal data are a crucial tool for examining the impact of recent rehousing and policing initiatives in the LA region. For example, both the city and county of LA have implemented encampment resolution programs in recent years, an effort to resolve longstanding encampments by moving residents into agency-provided motels or hotels [25,26]. While framed as a pathway to stability, it remains unclear whether these shelter and rehousing initiatives actually lead to better housing outcomes for those affected. In addition, some temporary sheltering and congregate housing models have been critiqued for producing what scholars characterize as “shelterization,” where institutional routines, surveillance, and loss of autonomy contribute to the erosion of identity, agency, and long-term housing readiness [57]. By surveying participants regarding their housing status and location each month, we will be able to track whether clients have been successfully rehoused and for how long, whether they have done so through provider support or self-resolution, and where they are rehoused. For respondents who remain unsheltered, we will be able to monitor both general health and well-being trajectories over time. These data will help inform local policy and guide the efforts of service providers and government agencies.

Finally, PATHS offers a flexible platform for evidence-based monitoring of other evolving issues in several key ways. First, the study’s recruitment and data collection strategies can be adapted to target specific populations of people experiencing homelessness, facilitating further investigations into policy and program development and evaluation. For example, across the United States, “street medicine” has emerged as a response to the inadequacy of traditional clinical models, attempting to deliver medical and psychiatric care directly to people experiencing homelessness in the spaces where they live, such as encampments, sidewalks, or temporary shelters [58,59]. These approaches reflect a growing recognition of the limitations of brick-and-mortar health care systems in reaching highly transient and marginalized people experiencing homelessness [59]. Using our existing recruitment strategy, we have recruited a sample of people experiencing homelessness engaged in street medicine services through interactions with health care providers. This sample will be compared with our representative sample to assess the effect of street medicine engagement on both health and housing outcomes. Our recurring data on a wide range of outcomes will provide valuable insights into the short-term and long-term effects of these programs, helping improve service distribution and informing future intervention strategies. Second, as part of our ongoing panel, we can update our surveys to include new questions, addressing emerging issues, initiatives, and interventions. For instance, in response to the wildfires in LA County that began on January 7, 2025, we added questions about the impact of wildfires on participants’ well-being to the monthly survey distributed on January 20, 2025. This rapid assessment will help understand the impact of wildfires and other extreme weather events on the unsheltered population, especially as such events become more prevalent, to guide interventions during these emergencies.

Looking beyond these specific topics, our survey platform can serve as a resource for other investigators, allowing them to invite respondents to participate in separate qualitative and quantitative studies aimed at gaining a deeper understanding of unsheltered individuals’ perceptions of and experiences with various policies and programs. Finally, the PATHS approach and technological platform can be extended to other populations considered highly vulnerable facing extreme, recurrent, and overlapping forms of marginalization. We have had discussions with research groups in other municipalities about extending the PATHS model and have begun to explore enrollment of samples in other populations facing overlapping and recurrent adverse exposures, such as precarious workers and people exiting prison [60,61].

### Limitations

Despite making significant advancements, the PATHS design has a few limitations. Most notably, PATHS requires mobile phone access at enrollment and an email address, raising concerns about generalizing findings from the cohort to the LA County unsheltered population, lacking digital access or literacy. While previous studies have shown that most of the unsheltered populations have access to a mobile phone at some point [34], the recruitment strategy may still miss individuals with transitory access or those who face difficulties maintaining a phone, such as those with severe cognitive impairments or more severe forms of neuropsychiatric disorders. In addition, individuals without internet-enabled phones or those who choose not to participate in research studies because of paranoia, suspiciousness, delusions, and cognitive decline may also be systematically excluded. This may result in the underrepresentation of the most marginalized subgroups of the unsheltered population and introduce selection bias by favoring participants with higher connectivity, potentially skewing findings toward individuals who are already somewhat engaged with services or supports. However, we are able to assess the extent of this sample selection criterion by comparing our respondent sample to the broader unsheltered population in LA County. This comparison allows us to create sampling weights that adjust for this bias in the analysis.

Furthermore, the use of web-based surveys in our study requires people to have internet access to participate, leading to challenges in keeping respondents engaged on a regular basis over a long time frame. Some level of attrition is inevitable, especially because respondents in our study population may lose their phones, run out of mobile data, or lack access to a computer with internet when surveys are distributed. To address these concerns and improve response rates, we introduced a “preferred contact mode” question in our monthly surveys, allowing respondents to choose whether they would like to receive their next survey via email or SMS text message. We also proactively send reminder emails to respondents who have not completed a survey in a while, encouraging them to update their contact information. By taking these steps, we aim to minimize attrition and ensure that our data remain as representative and reliable as possible over the course of the study.

Relatedly, 1 aspect of PATHS that serves as both a feature and a strength is the use of “Prefer not to answer” options on all questions. This trauma-informed approach ensures higher levels of survey completion, but at the cost of higher-than-usual missingness of individual survey items. As a result, many complex analyses of PATHS data require the use of multiple imputation or, at least, careful assessment of the comparability between completed cases and the full sample.

### Conclusions

PATHS provides a flexible and comprehensive approach to real-time monitoring of the unsheltered population in LA County, making it a valuable resource for future homelessness and health research and policy development. With PATHS now fully operational, we plan to expand our focus to explore critical areas affecting unsheltered individuals, such as the impact of wildfires and other natural disasters, as well as the effects of environmental stressors such as policing. In addition, we aim to evaluate the effectiveness of current interventions and support services tailored to this group considered vulnerable, assessing their impact on long-term housing stability and overall well-being. Through these efforts, we hope to contribute to more informed, evidence-based policies and interventions that can better address the evolving needs of unsheltered people experiencing homelessness.

## Appendix

Multimedia Appendix 1 Study enrollment via SMS text message and flyer.

Multimedia Appendix 2 Study enrollment web page.

Multimedia Appendix 3 Survey buffer messages.

## Abbreviations

DS: Demographic Survey
LA: Los Angeles
PATHS: Periodic Assessment of Trajectories of Housing, Homelessness, and Health Study
UCLA: University of California, Los Angeles

## Data Availability

The datasets generated or analyzed during this study are available from the corresponding author on reasonable request.
