# Supplementary figures and images for: Periodic Assessment of Trajectories of Housing, Homelessness, and Health Study (PATHS): Protocol for a Prospective Cohort Study of People Experiencing Homelessness
Source: JMIR Res Protoc. 2025 Sep 4;14:e74266. doi: 10.2196/74266 (PMC12447006; doi:10.2196/74266)

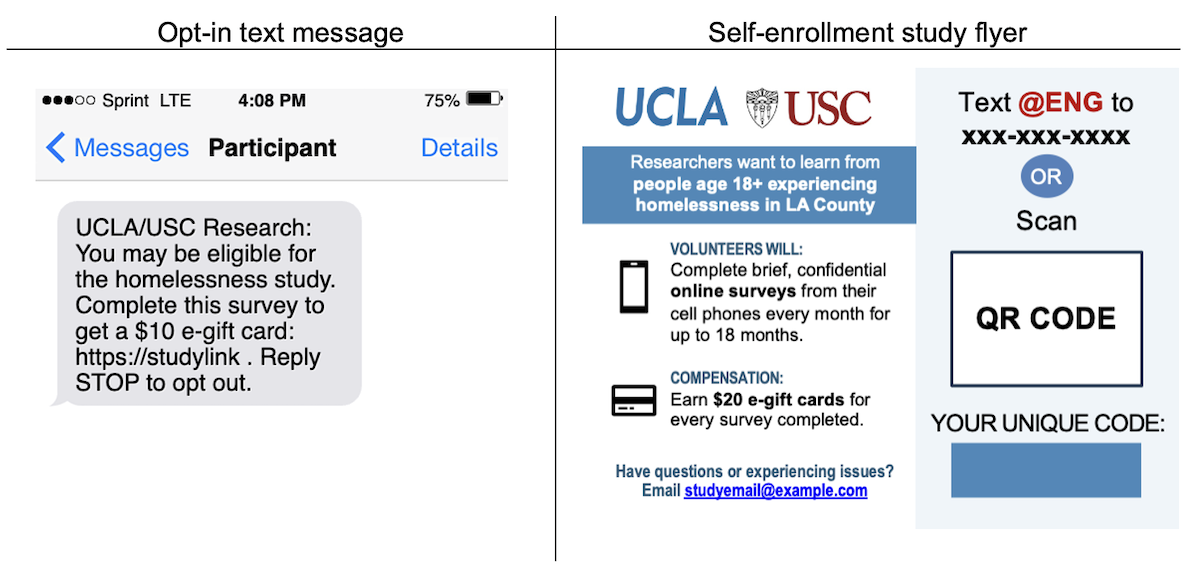

Supplement: Multimedia Appendix 1 [file resprot_v14i1e74266_app1.png]

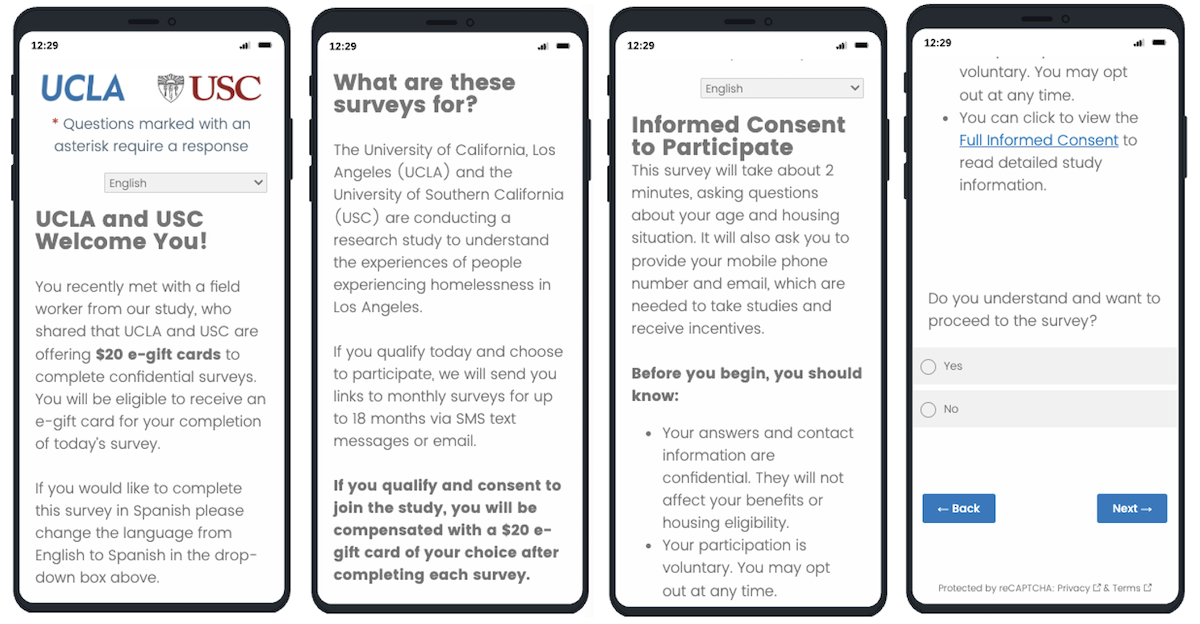

Supplement: Multimedia Appendix 2 [file resprot_v14i1e74266_app2.png]

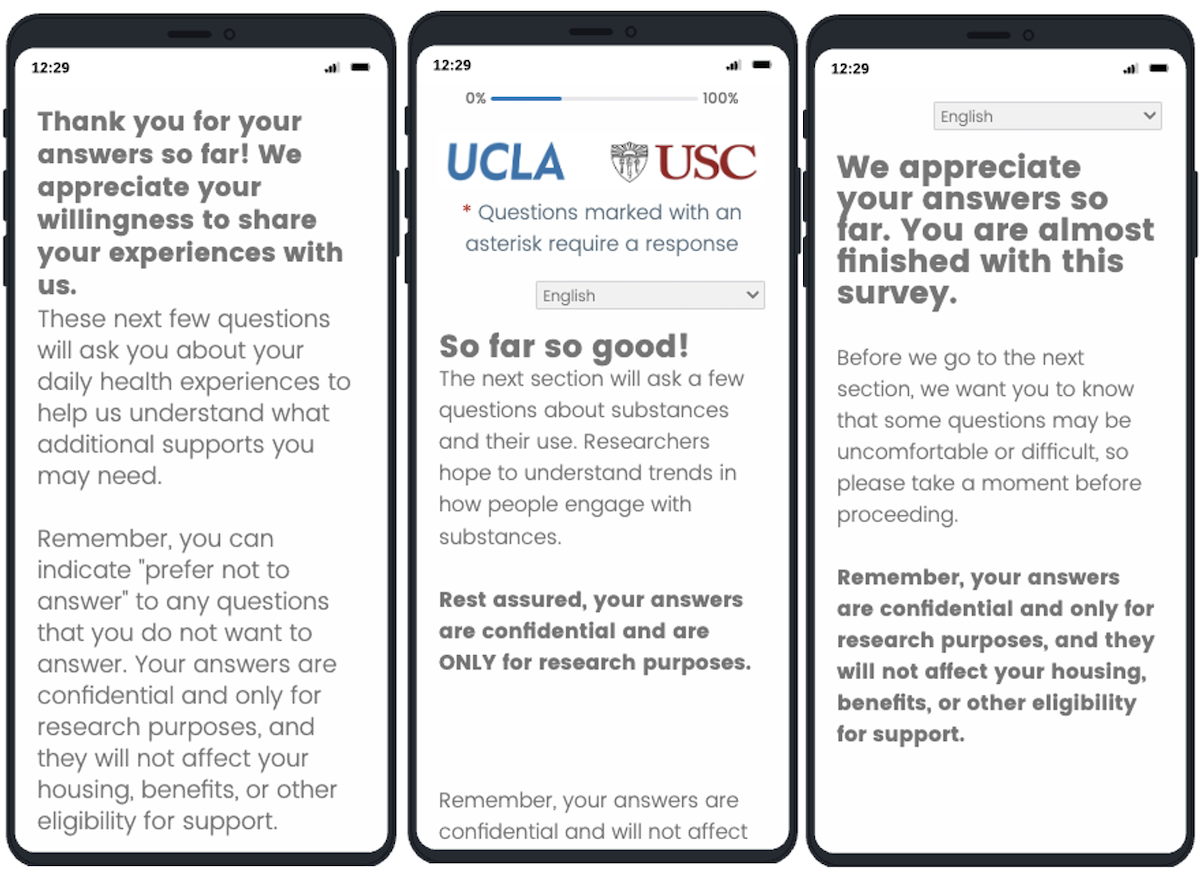

Supplement: Multimedia Appendix 3 [file resprot_v14i1e74266_app3.png]
